# Nursing interventions in preventing pressure injuries in acute inpatient care: a cross-sectional national study

**DOI:** 10.1186/s12912-023-01369-8

**Published:** 2023-06-12

**Authors:** Tarja Tervo-Heikkinen, Anniina Heikkilä, Marita Koivunen, Tiina Kortteisto, Jaana Peltokoski, Susanne Salmela, Merja Sankelo, Tuija Ylitörmänen, Kristiina Junttila

**Affiliations:** 1 Wellbeing Services County of North Savo, Kuopio University Hospital, PO Box 1711, Kuopio, FI70211 Finland; 2 Helsinki University Hospital and University of Helsinki. Group Administration, FI00029 HUS. Helsinki, PO Box 705, Helsinki, Finland; 3 Wellbeing Services County of Satakunta, Department of Nursing Science, Pori, Finland, and University of Turku, Sairaalantie 3, Turku, FI28500 Finland; 4 Wellbeing Services County of Pirkanmaa, Tampere University Hospital, Elämänaukio 2, PO Box 2000, Tampere, FI33521 Finland; 5 Wellbeing Services County of Central Finland, Hoitajantie 3, Jyväskylä, FI40620 Finland; 6 Wellbeing Services County of Ostrobothnia, Korsholmanpuistikko 44, Vaasa, FI65100 Finland; 7 Wellbeing Services County of South Ostrobothnia, Hanneksenrinne 7, Seinäjoki, FI60220 Finland; 8 Department of Nursing Science, University of Turku, Turku, Finland; 9 Finnish Institute for Health and Welfare, PO Box 30, Helsinki, FI00271 Finland; 10 Helsinki University Hospital and University of Helsinki, HUS Nursing Research Center, PO Box 442, Helsinki, FI00029 HUS Finland

**Keywords:** Pressure injury, Pressure ulcer, Risk management, Nursing assessment, Adult nursing

## Abstract

**Background:**

Several nursing interventions for pressure injury prevention have been identified, including risk and skin status assessment. The aim of this study was to explore prevention of pressure injuries in Finnish acute inpatient care. The data were collected on pressure injury risk and skin status assessments, repositioning, the use of support surfaces, preventive skin care, malnutrition risk assessment, and nutritional care.

**Methods:**

This multicentre, cross-sectional study was conducted in 16 acute care hospitals, excluding psychiatric care. Adult patients from inpatient care were recruited on the annual international Stop Pressure Ulcers Day in 2018 and 2019. Enrolment covered 6,160 participants in 503 units. Descriptive statistics were used to describe pressure injuries, risk assessments, and preventive nursing interventions. Cross tabulation, Pearson’s chi-square and Fisher’s exact tests were also used. Reporting follows the Strengthening the Reporting of Observational Studies in Epidemiology (STROBE) guidelines.

**Results:**

In all, 30% of the participants had their pressure injury risk assessed during the care, and for 19% within 8 h after admission. The same time limit in risk assessment was fulfilled for 16% of the participants with a pressure injury, and 22% of the participants using a wheelchair or being bedridden. A skin status assessment within 8 h after admission was conducted for 30% of all participants, and for 29% of the participants with a pre-existing pressure injury, and for 38% of the participants using a wheelchair or being bedridden. The risk of malnutrition was screened in 20% of the participants. Preventive interventions were targeted to participants with a pressure injury instead of patients with a high-pressure injury risk.

**Conclusion:**

This study adds evidence about pressure injury risk assessments and the implementation of preventive nursing interventions in Finnish acute care. Skin status and pressure injury risk assessments were irregularly conducted, and the outcome was not used by nurses to guide the implementation of preventive interventions. The results reveal the gaps in evidence-based nursing practice, which require further efforts to prevent pressure injuries. Improving the national focus on pressure injury prevention practice is critical for improving healthcare for our patients.

## Background

Preventing pressure injuries (PIs) is an important part of nurses’ work in hospitals and in communities. Pressure injuries arise from a failure of skin integrity due to unrelieved pressure and other forces, such as a bony area contacting external surfaces or a medical device causing pressure [1]. Although it is possible to prevent PIs in most cases, it requires proper preventative interventions [1]. Pressure injuries cause both direct and non-direct medical costs [2]; thus, treating PIs is more expensive than PI prevention [3]. Most importantly, PIs reduce the quality of life of patients [1, p11, 2], along with the quality of care [4]. Therefore, it is important for hospitals to invest in prevention efforts and early detection of PIs [3, 5]. An earlier study [6] identified that every tenth patient arriving at the hospital already had PIs or developed them within 36 h of admission. Thus, nurses and other health care personnel play a significant role in PI prevention. Moreover, healthcare managers have a crucial role in ensuring nurses’ access to evidence-based prevention methods, and that the prevention methods are in systematic use [7]. Hospital-acquired PIs (HAPIs) are globally one of the quality indicators of care [8], which lends to the conclusion that more attention should be given to PI monitoring and prevention in nursing.

At Odense University Hospital in Denmark, 17% of the patients (*n* = 427) had PIs in 2010. However, a study that took place six years later showed that the PI prevalence at two Danish hospitals dropped to 2% after implementing preventive interventions [9]. A recent study from Switzerland also showed a clinically significant improvement in statistics through evidence-based practice, and a significant reduction of HAPIs when using a multimodal improvement approach with measurement and feedback [10]. In a Swedish national patient-safety program from 2011 to 2020, the PI prevalence decreased from 17 to 11.4%, and HAPIs from 8.1% (year 2017) to 6.4% (year 2020) [11].

There are several risk factors for PIs, which are difficult to prioritise. The International Guideline [1, p39] identifies e.g., the following risk factors: activity and mobility limitations, moisture, sensory perception limitations, skin status, nutrition indicators, and older age. In addition, some patient groups are more vulnerable to PIs than others, e.g., individuals in the operating room, the critically ill, neonates and children [1, p28].

The International Guideline [1] recommends an immediate PI risk assessment with a reliable tool upon the patient’s admission to the healthcare services. The risk assessment should also be repeated each time there is a change in the patient’s condition. However, it should be noted that in addition to the risk assessment, risk factors need to be recognized to initiate interventions in order to mitigate the modifiable risk [1, p38]. In an earlier study, two-thirds of adult HAPI patients were identified as being at PI risk using the risk assessment [12].

According to the International Guideline [1], PIs usually occur over a bony prominence, such as heels, sacrum, scapula, ankles, elbows, ears, and the back of the head. Pressure injuries can also be related to different medical devices or other objects, e.g., respiratory devices, orthopaedic devices, urinary or faecal-collecting devices, repositioning devices, drains, tubes, and intravenous catheters and lines as well as restraints, stockings, bandaging, or different devices and objects without a medical function that are left in the patient’s bed or chair [1, p181].

It is important to monitor medical device related (MDR) Pls in hospitalised patients to identify those at risk and to prevent complications. A retrospective study from US and Canadian facilities found that the total MDR prevalence was 0.6% and the HAPI MDR prevalence was 0.46%. Most frequently, MDR PIs were associated with nasal oxygen devices (nose and ears), casts and splints, followed by CPAP or BiPAP masks [13]. Further, Kim and Lee [14] found that the incidence rate of MDR PIs was 0.09 cases per 1,000 days of hospitalisation, and MRD PIs accounted for 5.5% of all PIs.

According to the International Guideline [1], pressure injuries stage I are a prognostic factor for higher stage PIs. Each patient should go through a comprehensive skin and tissue assessment after admission, or after being transferred to healthcare services as a part of the risk assessment. A skin assessment should also be implemented prior to being discharged from healthcare services [1]. According to our earlier findings [15], PI risk and skin status assessments are of high importance for HAPI prevention. If neither assessment was performed, the odds of having HAPIs increased, especially for medical patients with a 15-fold increase in their risk of acquiring HAPIs.

A patient’s nutritional status plays a very important role in the wound healing process and PI prevention. Malnutrition predisposes patients to PIs and slows their healing as well as recovery from other diseases due to the body’s decreased ability to fight infections [16]. Therefore, the identification of malnutrition with a valid and reliable nutritional screening tool is important, as well as planning their nutritional care, when a patient is at risk of malnutrition [1].

When a patient is bedridden, his lying position and repositioning frequency are important aspects of nursing practice and the patient’s well-being. However, it is not clear how often patients need to be repositioned. The scoping review on repositioning frequencies revealed that only two studies had found significant differences when a patient was turned every three or four hours compared to every six hours [17]. The need for repositioning is also influenced by the type of support surface used for each patient [18]. Nevertheless, regular posture changes are important in PI prevention [1].

A large systematic review and meta-analysis [19] found moderate evidence that powered active and hybrid air surfaces most likely lessen PI incidences compared to standard hospital surfaces. However, the authors indicate that active air surfaces may not be as comfortable as standard hospital mattresses [19]. The mattresses play a major role in PI development, especially in intensive care units (ICUs), where a patient’s medical condition may be poor, and the patient is bedridden. A clinical observational study showed that PI incidence could be significantly reduced with the right choice of mattress [20].

In Finland, HAPI monitoring is not mandatory, so we do not have a clear picture of the PI prevalence or its prevention in our country. This article presents the results of the first national PI prevalence survey in Finland covering three-fourths of acute care hospitals in Finland. A report on PI and HAPI rates in acute inpatient care has been published earlier [15]. In this article, the focus is on PI prevention.

## Methods

### Aim

The aim of this study was to explore prevalence and prevention of pressure injuries in Finnish acute inpatient care. The objective was to give a representation of nursing practice in PI prevention for further development. The following research questions were addressed:

1) What is the PI prevalence in Finnish acute inpatient care; number, stages, and locations of community-acquired and hospital-acquired pressure injuries?

2) What are the compliance rates to guidelines for PI risk and skin status assessments in Finnish acute inpatient care in general and in specific patient groups?

3) What are the compliance rates to PI prevention interventions by nursing in Finnish acute inpatient care in general and in specific patient groups?

Reporting follows the Strengthening the Reporting of Observational Studies in Epidemiology (STROBE) guidelines.

### Design and setting

A multicentred cross-sectional study design was followed and conducted in Finnish acute inpatient care. In all, 16 out of 21 such organisations participated, and five of them were university hospitals and others were central hospitals.

### Participants and data collection

**Fig. 1:**
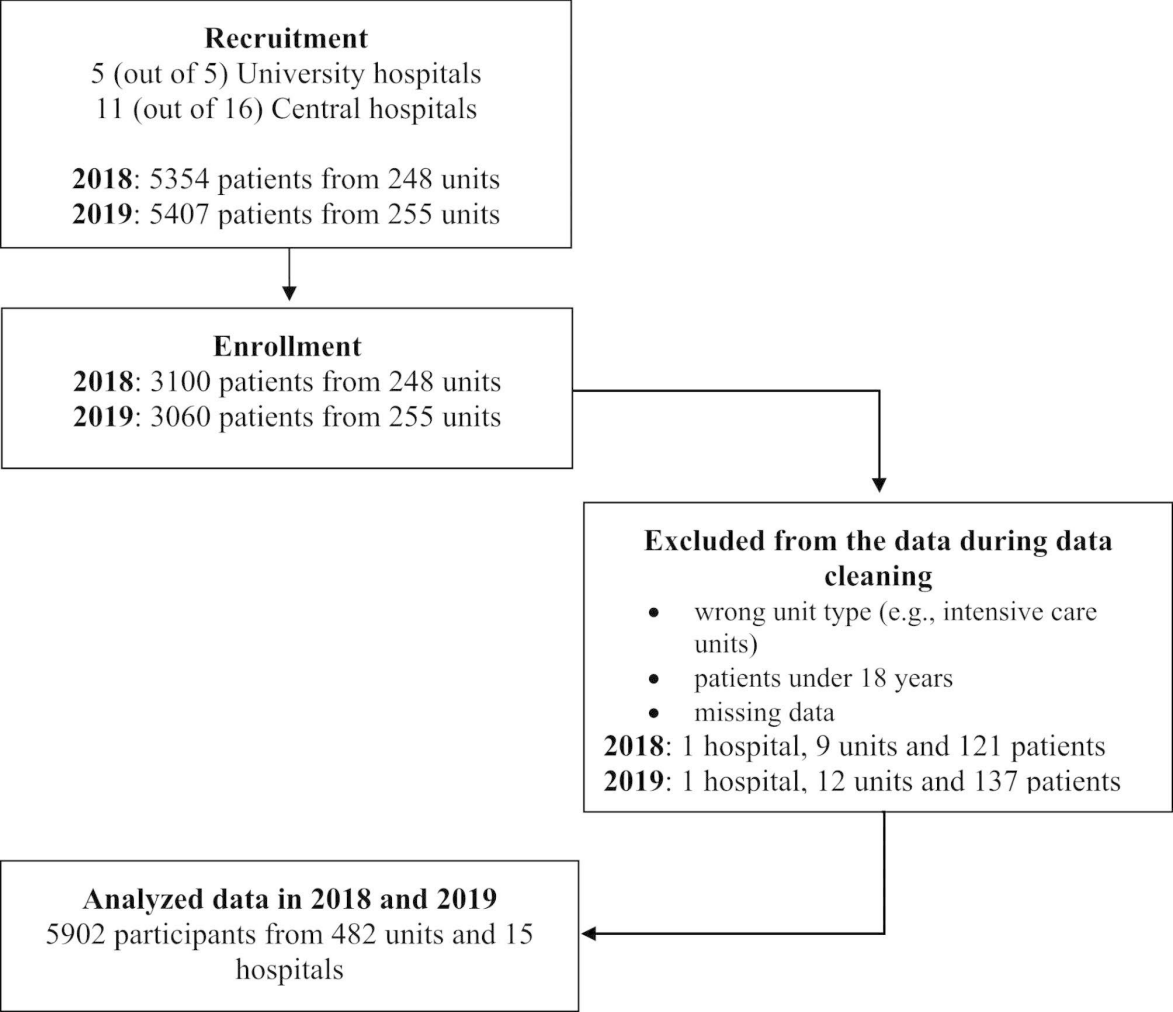
Research data and data cleaning

On the annual international Stop Pressure Ulcers Day in 2018 (15 November) and 2019 (21 November), all adults from inpatient units (excluding psychiatric care), emergency follow-up units, and rehabilitation units were recruited to participate in the study [15]. No exclusion criteria were set for the enrolment. In all, 6,160 participants from 503 units gave their consent to participate. The recruitment of participants is presented in Fig. 1.

The following research data were collected:

(1) Background information on the study units: a protocol to prevent PIs in use (yes/no), a protocol to conduct skin assessment within 8 h after admission in use (yes/no), a PI risk assessment instrument in use (e.g., Braden Scale [21], Norton Scale [22], no instrument in use), an instrument to assess the risk of malnutrition in use (e.g., NRS2002 [23], no instrument in use), number of hospital beds and inpatients on data collection days. This information was collected from the nurse managers.

(2) Data on skin condition and observed PIs: assessment of the skin condition of each study participant on the data collection day. The assessment was conducted either by a designated nurse or a nurse participating in the patient’s bedside care. The result of the assessment was recorded on a data-collecting form, along with the stages and anatomical locations of the observed PI(s). Pressure injuries were staged by using the quick guide for PI staging by the Finnish Wound Care Society, which followed the guidelines of the National Pressure Ulcer Advisory Panel, European Pressure Ulcer Advisory Panel, and Pan Pacific Pressure Injury Alliance (NPUAP/EPUAP/PPPIA) that were present at the time of the study [22]. According to the quick guide, the PI stages included stages I-IV, mucous membrane PIs, non-visible PIs, and further, unstageable, and suspected deep tissue injuries as one class. Additionally, for each PI it was recorded whether or not the PI was related to a medical device (the devices were not identified), and based on the electronic patient record, whether or not the participant had the PI at admission.

(3) Participant background information from the electronic patient record (EPR): age (in years), gender, mobility, mode of arrival to hospital (e.g., emergency), and surgical procedure (for surgical patients). Further, the consecutive number of the day(s) into the hospitalisation was documented, from which the data were collected. Additionally, the data included information on the PI risk assessment at admission (or later during the care) and its outcome, the assessment of the skin condition at admission (yes/no), and the outcome of the potential malnutrition risk assessment. The time limit of 8 h for assessments followed The Clinical Practice Guideline valid at the data collection time [24].

(4) Data on preventive interventions were collected from the nursing notes in the EPR: skin status assessment within 24 h (yes/no/contraindication documented), support surface in use (e.g. reactive support surface or mattress/contraindication documented), preventive skin care (e.g. dressings, such as foams or hydrocolloids/no need to use), repositioning (e.g. yes/no/contraindication documented), risk of malnutrition assessed within a week (yes/not assessed/not documented), and nutrition care at the moment of prevalence (e.g., not documented/parenteral nutrition/not per os).

Data sets 2–4 were collected either by designated nurses or nurses participating in patients’ bedside care. Organisational study coordinators entered the research data on a uniform data matrix. The participants’ identity codes were replaced with artificial codes. The data matrices were stored on a protected software, which uses mandatory user identification. The software has been designed and administered by one of the study organisations for sharing data-sensitive documents.

The data were cleaned as described in Fig. 1. The final analysed data comprised 5,902 participants in 482 units from 15 hospitals. The 15 study organisations are presented in Table 1.

**Table 1:** Description of study organisations (after data cleaning)

Study organisations, year 2019	Beds	LOS (mean)	Nursing staff, all	Eligible participants	Consent participants n (%)
**University Hospitals by location**					
Helsinki	2805	4.0	14,310	2477	1387 (56.0)
Kuopio	556	3.2	2962	749	409 (54.6)
Oulu	829	3.6	4157	966	476 (49.3)
Tampere	1226	3.8	4116	1460	897 (61.4)
Turku	981	3.2	4248	977	515 (52.7)
**Central Hospitals by location**					
Joensuu	865	4.3	1874	463	226 (57.5)
Jyväskylä	405	2.4	2209	333	201 (60.4)
Kotka	418	3.4	1453	272	131 (48.2)
Lahti	413	3.7	1775	552	347 (62.9)
Lappeenranta	215	4.1	3335	422	281 (66.6)
Pori	381	2.7	1886	496	307 (61.9)
Rovaniemi	276	3.0	1359	313	181 (57.8)
Savonlinna	97	2.9	325	147	62 (42.2)
Seinäjoki	365	3.4	1802	441	284 (64.4)
Vaasa	320	3.3	1443	380	198 (52.1)
**Total**	**10,152**	**3.4**	**48,273**	**10,448**	**5902 (56.5)**

Note: LOS = Length of stay (excluding psychiatric care)

### Data analysis

Descriptive statistics (frequencies and percentages) were used to describe demographic data, PIs, risk assessments and PI preventative interventions. Cross tabulation, Pearson’s chi-square (χ2) and Fisher’s exact tests were used to examine the statistical relationship between participant demographics (type of hospital, gender, age, mobility, mode of arrival) and observed PIs. Pressure injury risk assessment at admission (< 8 h), skin status assessment at admission (< 8 h), and the use of preventive interventions (repositioning, support surface, preventive skin care, assessment of malnutrition risk, nutrition care) were examined by Pearson’s chi-square (χ2) or Fisher’s exact tests in different subgroups: (a) participants with a PI versus participants without a PI, (b) participants with a high PI risk versus participants with a low PI risk, and (c) participants with limited mobility (wheelchair or bedridden patient) versus participants with no mobility problems.

P-values less than 0.05 were deemed statistically significant. For the analysis, the risk scores of the PI risk assessments were classified into two categories, e.g., the Braden Scale: ≤14 = high PI risk and ≥ 15 = low PI risk. [21]

Hospital acquired PI (HAPI) is defined as a PI acquired during the hospital stay. The pressure injury and HAPI rates for all PI/HAPI stages and stages II–IV were calculated by dividing the total number of patients with PI or HAPI by the total number of patients included in the analysis. For patients with more than one PI or HAPI, the most severe stage was used for the analysis.

The data were analysed with IBM SPSS Statistics for Windows (Version 25.0, Armonk, New York).

### Validity and reliability

Each study organisation had its own study coordinator responsible for data collection, and its conduction followed the uniform information material and data collection manual. However, the conduct of informing and training data collectors prior to data collection varied between the organisations, and their competence was not tested. The data collection also varied between organisations; the data were collected either by designated nurses or nurses participating in patients’ bedside care. The observed PIs were staged by following a national quick guide for PI staging as described earlier. Staging of PIs was conducted independently by a single nurse without further validation.

## Results

The participants’ demographic characteristics are presented in Table 2. More than half (62%) of the participants (n = 5902) were treated in university hospitals and medical units (54%). Most commonly, the participants were general medical patients (22%, n = 1267), gastroenterological or urological patients (12%, n = 687), orthopaedic patients (10%, n = 593), general surgical patients (8%, n = 478), and cardiac patients (7%, n = 431). The largest group of participants was 66–80 years old (39%); half (50%) had been admitted as emergency cases, and 64% had a surgical procedure during their hospitalisation.

**Table 2:** Demographic data of participants. Chi-square test or Fisher’s exact test were calculated between PI yes/no and variables

Variables	All (*n* = 5902)	Without PI (*n* = 5155)	With PI, all grades (*n* = 747)	χ2 -value (df)	P-value
**Type of hospital**					
University hospital	3684 (62.4)	3210 (62.3)	474 (63.5)	- (-)	0.55b
Central hospital	2218 (37.6)	1945 (37.7)	273 (36.5)		
Total	5902 (100.0)	5155 (100.0)	747 (100.0)		
**Gender**					
Male	3020 (51.3)	2625 (51.1)	395 (53.1)	-	0.31b
Female	2866 (48.7)	2517 (48.9)	349 (46.9)		
Total	5886 (100.0)	5142 (100.0)	744 (100.0)		
**Age, years**					
< 40	554 (9.5)	519 (10.2)	35 (4.7)	72.73 (3)	< 0.01a
40–65	1882 (32.3)	1700 (33.5)	182 (24.6)		
66–80	2258 (38.8)	1942 (38.2)	316 (42.6)		
> 80	1128 (19.4)	920 (18.1)	208 (28.1)		
Total	5822 (100.0)	5081 (100.0)	741 (100.0)		
**Mobility**					
Independent	3832 (65.3)	3549 (69.2)	283 (38.1)	311.22 (3)	< 0.01a
Needs assistance to move	1136 (19.3)	952 (18.6)	184 (24.7)		
Wheelchair	394 (6.7)	304 (5.9)	90 (12.1)		
Bedridden	511 (8.7)	324 (6.3)	187 (25.1)		
Total	5873 (100.0)	5129 (100.0)	744 (100.0)		
**Mode of arrival**					
Emergency care	2901 (49.6)	2524 (49.3)	377 (51.0)	109.85 (2)	< 0.01a
Elective	1765 (30.2)	1641 (32.1)	124 (16.8)		
Other	1187 (20.2)	949 (18.6)	238 (32.2)		
Total	5853 (100.0)	5114 (100.0)	739 (100.0)		

Note: PI = pressure injury, a = Pearson’s chi-squared test, b = Fisher’s exact test, χ2 -value = Pearson’s chi-square statistic value, df = degrees of freedom

The overall PI prevalence was 13% (*n* = 747, all stages), and 3% (*n* = 156) of the participants had at least one community-acquired PI at admission. Most of these participants were in the 60–80 age group (44%), used a wheelchair or were bedridden (55%), and were admitted to the hospital as emergency cases (46%). The hospital acquired PI (HAPI) rate (all stages) was 10% (*n* = 591). When including stages II–IV, it was 3% (*n* = 117). The detailed PI and HAPI rates and their risk factors have been reported earlier [15]. A pressure injury risk assessment (either < 8 h after admission or later during the care) was conducted for 30% of the participants (*n* = 1778). Of these participants, 30% (*n* = 530) were assessed as being at a high PI risk. The day on which the participant’s skin condition was assessed, took place (on average) on the seventh day of the hospital stay (range: 0–146 days).

Most PIs were at either stage I or II. Most HAPIs (44%) were in the lower limb (i.e., knee, leg, and foot, including heel and malleolus), as were most of the PIs at admission (39%). Of the HAPIs, 20% (*n* = 118) were medical device related (MDR) PIs, and most of these MDR PIs (35%) were located in the head area (Table 3).

**Table 3:** Stages and locations of HAPIs, medical device-related PIs and PIs at admission

	HAPI n (%)	Medical device-related HAPI n (%)	PI at admission n (%)
**Stages of HAPI/PI**	**n = 591**	**n = 118**	**n = 156**
Stage I	389 (65.8)	72 (61.0)	69 (44.2)
Stage II	143 (24.2)	36 (30.5)	53 (34.0)
Stage III	27 (4.6)	2 (1.7)	15 (9.6)
Stage IV	7 (1.2)	4 (3.4)	11 (7.1)
Unstageable PI	15 (2.5)	1 (0.9)	5 (3.2)
Mucous membrane PI	3 (0.5)	3 (2.5)	1 (0.6)
Non-visible PI	7 (1.2)	0	2 (1.3)
**Locations of HAPI/PI**	**n = 577**	**n = 105**	**n = 155**
Lower limb (knee, leg, and foot incl. heel and malleolus)	257 (44.5)	30 (28.6)	61 (39.3)
Head, face	54 (9.4)	41 (39.1)	10 (6.4)
Hand, arm, shoulder	41 (7.1)	12 (11.4)	4 (2.6)
Hip, buttock region (incl. sacrum)	211 (36.6)	16 (15.2)	72 (46.5)
Torso	14 (2.4)	6 (5.7)	8 (5.2)

Note: HAPI = hospital-acquired pressure injury, PI = pressure injury. For patients with more than one PI or HAPI, the most severe stage was used for the analysis

Nurse managers reported a protocol of systematic skin status assessments within eight hours of admission to be utilised in 49% of the study units (range by organisation from 6 to 100%). According to the data, 30% (*n* = 1746) of the participants had their skin status assessed within 8 h of admission (range by organisation from 10 to 51%). A pressure injury risk assessment instrument was reported to be utilised in 66% of the study units (range by organisation from 0 to 100%). The risk was assessed for 19% (*n* = 1121) of the participants less than 8 h after admission (range by organisation from 1 to 59%). Correspondingly, the malnutrition risk screening instrument was reported to be utilised in 70% of the units (range by organisation from 0 to 100%). According to the data, the risk of malnutrition was screened in 20% (*n* = 1106) of the participants (range by organisation from 0 to 49%).

Statistically, the PI risk assessment at admission was significantly more frequently conducted for participants without PI (*p* = 0.04) and with limited mobility (*p* = 0.01) than their peers. The risk assessment at admission was not carried out for 60% of the participants, which were later during the care identified to have a high PI risk, for 84% of the participants with a pre-existing PI and for 78% of the participants using a wheelchair or being bedridden (Table 4).

**Table 4:** Skin status and PI risk assessments by subgroups; frequencies, percentages, and *p*-values of the Fisher’s exact test

Assessment		All participants (n = 5902)	With PI, all stages (*n* = 747) n (%)	Without PI (*n* = 5155) *n* (%)	*p*-value	Wheelchair or bedridden (*n* = 905) n (%)	Independent or needs assistance to move (*n* = 4986) n (%)	*p*-value
**Skin status < 8 h after admission**	Yes	1746 (29.6)	217 (29.0)	1529 (29.7)	0.73	346 (38.2)	1391 (28.0)	< 0.01
	No	4156 (70.4)	530 (71.0)	3626 (70.3)		559 (61.8)	3577 (72.0)	
	Total	5902 (100.0)	747 (100.0)	5155 (100.0)		905 (100.0)	4968 (100.0)	
**PI risk < 8 h** **after admission**	Yes	1121 (19.0)	121 (16.2)	1000 (19.4)	0.04	199 (22.0)	917 (18.5)	0.01
	No	4781 (81.0)	626 (83.8)	4155 (80.6)		706 (78.0)	4051 (81.5)	
	Total	5902 (100.0)	747 (100.0)	5155 (100.0)		905 (100.0)	4968 (100.0)	

Note: PI = pressure injury

The skin status assessment at admission was statistically significantly more frequently conducted for participants with a high PI risk (*p* < 0.01) and with limited mobility (*p* < 0.01) than their peers. The skin status assessment was not carried out for 53% of the participants with a high PI risk, for 71% of the participants with a pre-existing PI and for 62% of the participants using a wheelchair or being bedridden (Table 4).

The statistics also show that preventive interventions (repositioning, support surface, preventive skin care) were significantly (*p* < 0.01) more frequently conducted for participants with a PI and participants using a wheelchair or being bedridden compared to their peers. A malnutrition risk assessment was done more comprehensively for the high PI risk participants (*p* < 0.01) and PI participants (*p* = 0.01) than their peers. Statistically, nutrition care was significantly more frequently implemented for participants with a PI (*p* < 0.01) and with limited mobility (*p* < 0.01) than their peers. For participants with a PI or limited mobility, the statistics show that preventive interventions were used significantly more frequently than their peers. Table 5 describes the usage of preventive interventions by subgroups.

**Table 5:** Preventative nursing interventions by subgroups; frequencies, percentages, and p-value of the Chi-square test or the Fisher’s exact test

Preventive interventions n (%)	High PI risk (n = 530) n (%)	Low PI-risk (n = 1248) n (%)	χ2 -value (df)	p-value	Patient with PI, all grades (n = 747) n (%)	Patient without PI (n = 5155) n (%)	χ2 -value (df)	p-value	Wheelchear or bedridden (*n* = 905) n (%)	Independent or needs assistance to move (n = 4986) n (%)	χ2 -value (df)	p-value
**Repositioning**												
Yes	66 (12.9)	170 (14.1)	8.50 (2)	0.01a	214 (30.2)	506 (10.2)	301.89 (2)	< 0.01	469 (55.1)	249 (5.2)	1680.77 (2)	< 0.01a
No	60 (11.7)	203 (16.8)			176 (24.9)	776 (15.6)			149 (17.5)	801 (16.7)		
Unnecessary for the patient	387 (75.4)	836 (69.1)			318 (44.9)	3677 (74.2)			233 (27.4)	3746 (78.1)		
Total	513 (100.0)	1209 (100.0)			708 (100.0)	4959 (100.0)			851 (100.0)	4796 (100.0)		
**Support surface**												
Hospital basic bed foam mattress	320 (61.5)	633 (51.4)	23.79 (2)	< 0.01a	298 (41.3)	2761 (55.2)	152.67 (2)	< 0.01	340 (38.3)	2704 (56.1)	580.39 (2)	< 0.01a
Reactive support surface / mattress	159 (30.6)	528 (42.9)			315 (43.6)	2026 (40.5)			345 (38.9)	1990 (41.3)		
Active support surface / mattress	41 (7.9)	70 (5.7)			109 (15.1)	217 (4.3)			202 (22.8)	124 (2.6)		
Total	520 (100.0)	1231 (100.0)			722 (100.0)	5004 (100.0)			887 (100.0)	4818 (100.0)		
**Preventive skin care**												
No need	236 (81.9)	477 (80.7)	0.56 (2)	0.76a	161 (41.9)	2112 (88.0)	507.01 (2)	< 0.01	228 (47.2)	2038 (88.9)	464.03 (2)	< 0.01a
Dressings. e.g., foams, films, hydrocolloids	25 (8.7)	49 (8.3)			105 (27.4)	83 (3.5)			91 (18.8)	97 (4.2)		
Heel protector devices, dressings, other pads	27 (9.4)	65 (11.0)			118 (30.7)	204 (8.5)			164 (34.0)	157 (6.9)		
Total	288 (100.0)	591 (100.0)			384 (100.0)	2399 (100.0)			483 (100.0)	2292 (100.0)		
**Assessment of malnutrition risk**												
Yes	206 (40.5)	346 (29.0)		< 0.01b	57 (66.3)	301 (33.5)		< 0.01	178 (20.7)	924 (19.9)		0.56b
No	303 (59.5)	849 (71.0)			29 (33.7)	597 (66.5)			680 (79.3)	3726 (80.1)		
Total	509 (100.0)	1195 (100.0)			86 (100.0)	898 (100.0)			858 (100.0)	4650 (100.0)		
**Nutrition care**												
Oral nutrition with hospital food or enteral feeding	376 (82.8)	890 (82.5)	0.03	0.87b	441 (69.9)	3527 (85.7)	99.93	< 0.01	530 (70.4)	3422 (86.1)	113.38	< 0.01b
Oral nutrition supplements, energy-dense nutrition or enteral feeding or parenteral nutrition	78 (17.2)	189 (17.5)			190 (30.1)	588 (14.3)			223 (29.6)	554 (13.9)		
Total	454 (100.0)	1079 (100.0)			631 (100.0)	4115 (100.0)			753 (100.0)	3976 (100.0)		

Note: PI = pressure injury, PI risk = latest evaluation of PI risk, a = Chi-square test, b = Fisher’s exact test, χ2 -value = Pearson’s chi-square statistic value, df = degrees of freedom

## Discussion

The aim of this study was to explore prevalence and prevention of pressure injuries in Finnish acute inpatient care. According to our results, there was an overall PI prevalence of 13% and a HAPI rate of 10% in all stages, and PI prevalence of 4% and HAPI rate of 3% for stage II or above. The findings are similar to the results of previous European studies [25]. 3% of the participants had at least one community-acquired PI. In earlier studies, the corresponding portions have varied from 10 to 15% [6].

In this study, PIs were mostly located in the lower limb area and in the hip/buttock area. In earlier studies, most of them have been found to be in the sacrum [11, 12, 25–27] trochanter [26], pelvic area [13] and heel [11, 25, 27]. In this study, due to the low number of PIs, we had to combine some anatomic locations for analysis and reporting. For example, in our data locations of a knee, leg, foot (including heel) and malleolus were integrated into lower limb. Therefore, the comparison with earlier studies is difficult. However, the biggest group (16%) within the study participants suffered from diseases of the circulatory system, and they represented 18% of the HAPI patients [15]. The underlying diagnosis may explain the slight differences with earlier studies.

Additionally, medical device related (MDR) PIs were identified in 20% of all HAPIs, corresponding to an incidence of 2%. Most of these were in the head or lower limb. The results differ from findings in the US and Canada, where the MDR PI prevalence was 0.46% [13], i.e., clearly lower than it is in this study. It is important to acknowledge that patients wearing any medical device (for example, oxygen devices, CPAP masks, nasogastric tubes, endotracheal tubes, or casts) during their hospital stay are at risk of getting a HAPI. According to the European Union regulations (EU 2017/745) hospital beds are identified as medical devices. It may be that in this study, many PIs located in the lower limb area were categorised as medical device related PIs. In this study we did not identify medical devices behind MDR PIs.

In the literature, several nursing interventions for PI prevention have been identified, including assessment and prevention protocols [1, 11, 18], the use of support surfaces [11, 12], repositioning [11, 17, 18] and pressure ulcer bundles [9, 10]. In this study, data were collected on PI risk and skin status assessments, repositioning, the use of support surfaces, preventive skin care, malnutrition risk assessment, and nutritional care.

Of all participants, only one-third had their PI risk assessed during their hospital stay. Of those, 35% had their PI risk assessment carried out in less than eight hours after their admission. The variation between study organisations was wide (from 10 to 51%). Furthermore, one-third of the patients that already had a PI, and one-third of the patients with limited mobility had their PI risk assessed within eight hours of admission. The results are similar to a review carried out in Scandinavia, Iceland, and Ireland, where the PI risk assessment was found to be irregular and influenced by the organisational context [28]. At the data collection time, The Clinical Practice Guideline [24] recommended the PI risk and skin status assessments to be carried out within 8 h after admission. In current guideline, the exact time limit has been excluded [1].

The skin status assessment was carried out for nearly one-third of the participants already having a PI, and for two-fifths of the participants using a wheelchair or being bedridden (Table 4). Thus, the protocols [e.g., 1] for preventing PIs, including the PI risk and skin status assessments, are not adequately implemented in Finnish acute inpatient care.

Repositioning was carried out for half of the participants with limited mobility and those that were bedridden (Table 5). Systematic repositioning has been identified as a crucial part of their care, especially for these patients [1]. Additionally, the support surfaces were used irregularly (Table 5). Two-thirds of participants with a high PI risk and two-fifths of participants with a PI had a basic hospital foam mattress on their bed on the prevalence day. A third of participants with limited mobility also had a basic mattress. Active support mattresses were rarely used, which may be due to inadequate procurement [21].

This study found that preventive skin care (such as protective dressings and heel protection products) was rarely used for participants with a high PI risk, along with more than half of the participants in a wheelchair or bedridden patients. The results are consistent with an earlier study by Bredesen et al. [27] that found that many PI risk patients did not receive adequate preventive skin care, such as repositioning and support surfaces. Remarkably, in a study by Källman et al. [11], the usage of preventive interventions, such as pressure-reducing mattresses, increased from 74 to 96% in nine years during a national patient-safety program.

According to the current results, there is a big difference between the nurse manager’s report of implemented nursing protocols in the unit and the actual nursing practices. The biggest difference was found in the malnutrition risk screenings. According to the nurse managers, it was utilised in 70% of the units, but the screening was carried out only for a fifth of the participants. The malnutrition risk screening was not performed systematically for all PI risk participants and participants with reduced mobility. In turn, nutritional care was given to one-third of the participants with a PI or reduced mobility. Shortages in nutritional screenings and monitoring nutritional care in acute care hospitals have also been reported in other studies. For example, in a study by Bonetti et al. [29], a large percentage of hospitalised patients were at risk of malnutrition, and one-fifth of the patients were malnourished in acute care hospitals. Additionally, a bundle of care interventions that includes a malnutrition assessment is used in preventive care [10] and has been shown to be effective in PI prevention [9]. Based on the current results, nutritional routine screenings and additional monitoring in nutritional care are recommended for adoption in all inpatient units in Finland.

To summarise the results, the preventive interventions were targeted to participants with a PI instead of patients at a high PI risk. This suggests that the PI risk assessment did not lead to preventive interventions until the PIs developed. Therefore, the systematic skin assessments and PI risk assessments are crucial at admission [15] to adequately target the preventive interventions. Continuous skin assessment is also crucial in preventing PIs from progressing to a prolonged hospital stay. At the same time, nurse managers’ perceptions of care protocols in use were much more positive than their actual usage according to the results. Mäkinen et al. [30] conducted a study in Finland, in which they noticed that nurses do not see themselves as responsible for PI prevention. In this study, we did not explore nurses’ competence or attitudes towards PI prevention, but the results indicate low compliance with care protocols. The results suggest a continuous discussion is warranted in units about care protocols, their rationale, and their value for patients, the staff and the organisation. It may also be asked if the fundamental aspects of nursing care have been replaced with more technical to-do-lists, either due to nurse shortages or other factors related to leadership.

The reported results show that there is a shortage in implementing HAPI prevention in Finnish acute care hospitals. This may be partly explained by the state governance over health care services in Finland. Fundamentally, the law requires evidence-based and safe patient care [31]. At the same time, quality indicators, such as PI and HAPI prevalence, are not nationally registered and reported. Systematic and transparent reporting of PI rates is needed for continuous quality improvement in health care organisations, and also for the population for making informed decisions when choosing a care facility. Further, there are no national penalties to decrease hospital-acquired adverse events (such as PIs) as there are in some countries, e.g., the USA [32] and Australia [33]. Therefore, it may be that acute care hospitals totally miss or randomly use PI prevention protocols; consequently, the monitoring of systematic PI risk and skin status assessments, and PI prevalence and incidence varies between organisations. According to Källman et al. [11], a national patient-safety program in Sweden has echoed on PI prevalence and the occurrence of prevention strategies.

The findings of this study identify educational and research opportunities within Finnish acute care for PI prevention. There is a need for further focus on improving how the PI preventive interventions are implemented in practice. The International Guideline [1] clearly indicates the type of assessments and interventions required, as well as their timing, but it is evident they are not always ideally implemented. Thus, it is important to explore and identify barriers and facilitators in implementing PI prevention in hospitals. Exploring which PI assessments and prevention methods have been used effectively in other acute inpatient care settings is recommended as is the use of bundled care [10]. Further, exploring nurses’ competence and attitudes towards PI prevention would be beneficial.

This is the first national PI study in Finland that includes big data from fifteen acute care hospitals, covering 71% of such hospitals. At this stage, the follow-up and reporting of PIs is not mandatory in our country; due to not having any previous data, we could not compare our sample and PI findings. The purposive sampling was limited to adult patients in defined unit types, excluding the psychiatric units. Thus, this study provides merely a snapshot of the PI and HAPI prevalence, and preventive nursing interventions at the data collection points in the defined population and context.

We managed to enrol about two-thirds of eligible patients in the study (variation by organisation from 42 to 67%), which indicates somewhat unsuccessful recruitment process in the study units. This may be due to the nursing staff’s unfamiliarity or unwillingness to engage in participant enrolment in a fast-paced clinical practice. In addition, the process of written informed consent may have been too heavy for elderly patients or patients in a less healthy condition. Although we had a common manual with detailed instructions for data collection, it did not give advice on patient recruitment.

Data collection was supported by a common manual, as was the classification of PI stages under a national guideline. Valid tools such as the Braden Scale were used in PI risk assessment. However, the organisation of data collection varied between hospitals, and the data were collected either by designated nurses or nurses participating in patients’ bedside care. Their competency was not tested. Thus, variability in knowledge may have contributed to inaccurate PI staging.

In our data, the information on possible care in an intensive care unit was not collected, although it obviously influenced the development of PIs and MDR PIs. The pressure injury risk was documented and available from only 30% of the participants, which affected the analysis. Thus, the findings of this study on PI risk-related interventions are limited.

## Conclusion

This study adds evidence about PI risk assessments and the implementation of preventive nursing interventions in Finnish acute care. Skin status and PI risk assessments were irregularly conducted, and the outcome was not used by nurses to guide the implementation of preventive interventions, as they were targeted to participants with a PI instead of patients with a high PI risk. For the high PI risk participants, the most frequent preventive nursing intervention was malnutrition risk assessment, and the least frequent intervention the use of active support surface or mattress. The results reveal the gaps in evidence-based nursing practice, which require further efforts to prevent PIs. Improving the national focus on PI prevention practice is critical for improving healthcare for our patients.

## Data Availability

The datasets generated and/or analysed during the current study are not publicly available due to participants’ privacy but are available from the corresponding author on reasonable request.

## Abbreviations

*BiPAP*: Bilevel positive airway pressure
*CPAP*: Continuous positive airway pressure
*EPR*: Electronic patient record
*EPUAP*: The European Pressure Ulcer Advisory Panel
*HAPI*: Hospital Acquired Pressure Injury
*ICU*: Intensive care unit
*LOS*: Length of hospital stay
*MDR*: Medical device related
*NPIAP*: National Pressure Injury Advisory Panel
*NRS2002*: Nutrition Risk Screening 2002
*PI*: Pressure Injury
*PPPIA*: Pacific Pressure Injury Alliance
*STROBE*: Strengthening the Reporting of Observational Studies in Epidemiology
